# The role of position in consensus dynamics of polarizable networks

**DOI:** 10.1038/s41598-023-30613-z

**Published:** 2023-03-09

**Authors:** Helge Giese, Felix Gaisbauer, Nico Gradwohl, Ariana Strandburg-Peshkin

**Affiliations:** 1 Charité – Universitätsmedizin Berlin, Berlin, Germany; 2 University of Konstanz, Konstanz, Germany; 3 Max Planck Institute of Animal Behavior, Konstanz, Germany

**Keywords:** Psychology, Human behaviour

## Abstract

Communication constraints often complicate group decision-making. In this experiment, we investigate how the network position of opinionated group members determines both the speed and the outcome of group consensus in 7-member communication networks susceptible to polarization. To this end, we implemented an online version of a color coordination task within experimentally controlled communication networks. In 72 networks, one individual was incentivized to prefer one of two options. In 156 networks, two individuals were incentivized to prefer conflicting options. The network positions of incentivized individuals were varied. In networks with a single incentivized individual, network position played no significant role in either the speed or outcome of consensus decisions. For conflicts, the incentivized individual with more neighbors was more likely to sway the group to their preferred outcome. Furthermore, consensus emerged more slowly when the opponents had the same number of neighbors, but could not see each other’s votes directly. These results suggest that the visibility of an opinion is key to wielding group influence, and that specific structures are sufficient to run communication networks into polarization, hindering a speedy consensus.

## Introduction

Both animal and human groups often need to find mutual agreement to effectively exploit their environment^1^ or to fulfill social needs^2^. For example, cohesively moving animal groups must come to consensus on when and where to move^3^, while human groups must come to consensus on topics ranging from where to go for lunch to political decisions. Consensus typically must be reached via communication among group members. Yet, in many cases communication is constrained such that individuals only communicate directly with specific neighbors rather than globally with all group members^4^. In animal groups, individuals are typically only able to observe and interact with group members who are sufficiently close to them in space^4^. In human groups, communication links are often also shaped by physical proximity, but especially with the rise of the internet and social media can also reflect other social, economic, or political ties. The resulting communication networks for both animal and human groups can vary dramatically in their efficiency at spreading information and behavior, depending on the structural arrangement of communication ties^5–8^ and the distribution of opinions across network positions^9–14^. In this study, we empirically investigate how communication network structures interact with the opinions of individuals populating the network to determine the speed and outcomes of group-wide consensus decisions.

To reach a consensus, it is often crucial for groups to effectively reconcile divergent opinions, information, and preferences^15^. In some cases, individuals with a preference for a particular outcome may successfully coordinate the diffusion of behaviors, resulting in the group selecting their preferred outcome^16^. Such opinionated individuals can also play a key role in determining the speed of consensus^17,18^. However, when two or more individuals have conflicting preferences, not only can such conflict slow down the consensus finding process, but it can also qualitatively change how a consensus is reached^18^: While increasing the density of communication channels (i.e. network ties) can often speed up group decision-making processes, this effect can be mitigated under conflicts of interest^18^. Likewise, the number and position of unopinionated individuals becomes more relevant in conflict scenarios^18,19^.

### Differences in influence based on network degree vs. information centrality

Prior theoretical and empirical work suggests that individuals more centrally located in a group’s communication network may play an outsized role in facilitating the coordination needed for consensus. However, little is known about the specific function of central individuals in consensus decision making. The different possible mechanisms underlying how an individual’s network position translates into influence may be best captured by the different definitions of network centrality.

On the one hand, individuals may exert more influence on the group outcome by displaying their opinions more visibly to others using their higher number of contacts, as captured by the notion of degree centrality^20^. In this vein, Kearns et al.^13^ showed that conflicting networks designed to have higher degree differences were considerably faster, and leaned toward opinions held by individuals with higher degrees. Other studies have shown that tweaking the degrees of opponents may even lead to false perceptions of which opinions are held by the majority, ultimately biasing consensus decisions^10,11^.

On the other hand, opinionated individuals may also influence the group by selecting which information they transmit^21–23^. This ability to influence networks through spreading information is reflected in centrality measures that capture more global information than degree centrality, for example information centrality—defined as the harmonic average of the inverse of all communication path lengths involving a node^20^. In this regard, Fitch and Leonard^17^ showed via theoretical models that individuals high in this form of centrality spread information most efficiently and are the most effective at leading groups to come to consensus on their preferred outcomes. Similar effects linked to these types of centrality can be observed in coordination games^24,25^. Yet, because these different types of centrality influence are typically conflated in random or naturally observed networks^26^, their potentially different roles in finding consensus, especially in conflict scenarios, remains unknown.

### Polarization of opinions

Natural networks typically show clusters of opinions^27^, also referred to as polarization. Such polarization can arise both due to the tendency to form ties to like-minded individuals (homophily) and due to the tendency to adopt the opinions of social ties. In this study specifically, we define polarization as the group average of the difference between the proportion of individual agreement to directly tied neighbors and non-neighbors (for further details and mathematical operationalization see Section 3 of the Supplement). Polarized opinion networks are widely regarded as problematic, as polarization slows down consensus formation and increases the likelihood of deadlocks^11,13^.

It is unclear whether problems in finding consensus primarily stem from disadvantageous distributions of individual opinions in a network, or whether polarization and associated problems can also be provoked merely by the setup of specific communication network structures. Centola^6^ indicated it may be beneficial to have reinforcing, clustered ties for the spread of information. In cases of conflict, however, densely-tied neighborhoods that receive little and indirect feedback from outside may quickly run into false majority beliefs and therefore be in danger of polarization and deadlocks^11^. As such, it may be crucial for the avoidance of such states that a potential conflict of interest is directly and not indirectly experienced, with a network structure allowing people with contradicting opinions to directly communicate.

### Current study

**Figure 1:**
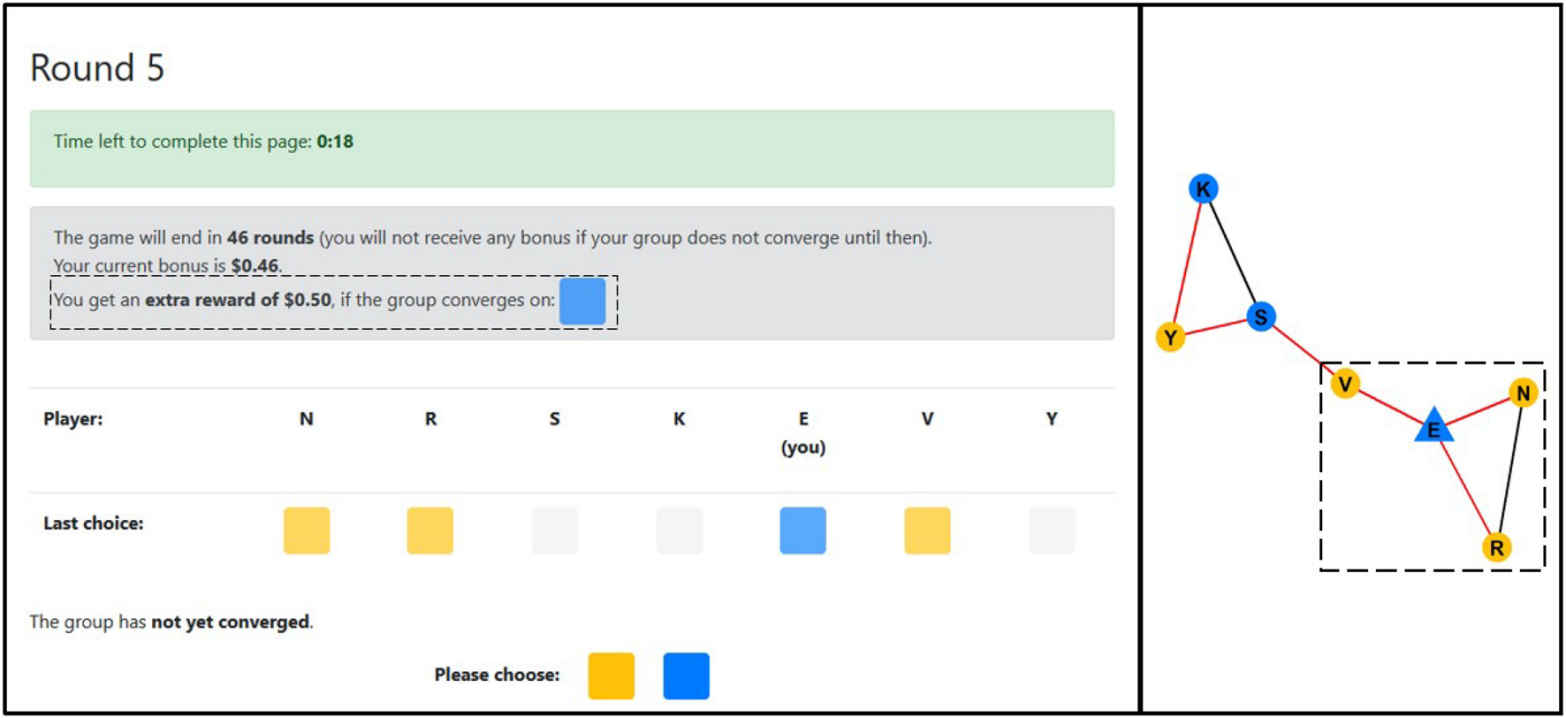
An example task screen (left) and the underlying network structure of the task (right). Left: A horizontal array of colored boxes represents the choices made in the last round. The choices of players not neighboring in the network are greyed out. In the example, players V, N, and R are visible to focal player E, who has been assigned the target to sway the group to blue. Only individuals incentivized to be opinionated about a specific target color saw the information about the extra reward and target color (dashed box). Right: Visualization of the underlying network structure. Note that this information was NOT visible to players. Node colors represent players’ choices in the last round, and the triangle node shape represents the incentivized individual.

In this study, we explore how the positions of people with specific preferences in a communication network affect consensus formation in a color coordination game^13,18^, in which all members of a network have to coordinate to eventually agree on one of two colors by observing others color choices and voting within 50 trials. We study two scenarios: a “leadership” scenario where the group contains a single individual (henceforth “leader”) additionally incentivized to prefer one of the colors, and a “conflict” scenario where the group contains two individuals with conflicting incentivization (henceforth “opponents”). In particular, we aim to compare the effects of degree vs. information centrality and direct vs. indirect conflict on the speed and outcomes of consensus by randomly allocating the opinionated individuals to a position in 7-individual dumbbell-shaped communication network (Fig. 1) and testing whether any positioning will be superior for the consensus outcome or speed. Extending on classic group experiment structures, this dumbbell network allows us to explicitly contrast different centrality definitions, compare indirect and direct conflict, and to emulate a small-world structure^25,28^.

We expect to replicate the finding that conflict slows down consensus^18^ and that under conditions of leadership, networks will form a consensus faster if that leader is more centrally located (e.g.^12^). We also test whether degree central (in particular, S and E in Fig. 1) or information central leaders (in particular, V in Fig. 1) and opponents wield more influence over group outcomes (see Fig. S1 for detailed differences between network positions in a set of centrality measures). In addition, we expect that direct conflicts between opponents (i.e. when two individuals with opposing incentives are directly tied/neighbors) should be resolved quickly because opinionated individuals experience imminent deadlocks first-hand. In contrast, in cases of indirect conflict (i.e. when opposing individuals are apart and cannot see one another directly), consensus should require more time due to the need for information to diffuse along neighborhoods of followers before reaching opponents.

## Results

### Speed of consensus in the two scenarios

**Figure 2:**
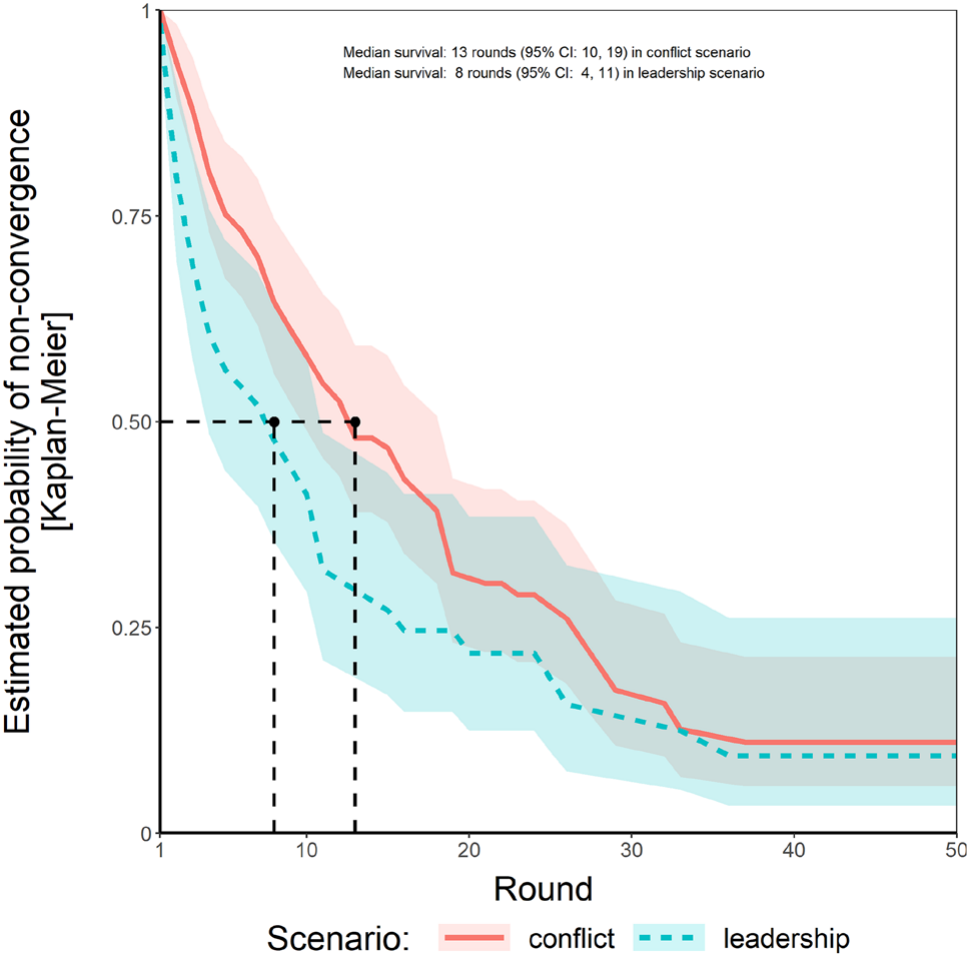
Consensus speed by scenario. Shaded areas represent 95% CI.

To some degree, we could replicate previous findings that convergence is slower under conflict compared to leadership, yet the effect was marginally significant only (*log-rank-*χ^2^ (*N* = 228, *df* = 1) = 3.64, *p* = 0.056, Fig. 2).

### Positional influence of a single opinionated individual (leadership)

In replication of Gaisbauer et al.^18^, we find that a single, opinionated individual tends to dictate group choice, thus effectively exerting leadership. 35 out of the 41 converged leadership networks agreed on the leader’s target color (85% of the converged networks involving a leader; 95% CI [71%, 94%], p_binom(41, 0.5)_ < 0.0001). The leader’s position in the network neither significantly predicted the outcome of consensus formation (χ^2^(*N* = 72, *df* = 6) = 1.39, *p* = 0.967) nor the speed of the process (*log-rank-*χ^2^(*N* = 72, *df* = 2) = 1.34, *p* = 0.511).

### Positional influence of opposing opinionated individuals (conflict)

Under conflict of interest, most networks converged and successfully averted deadlocks (78 out of 84 non-dropped networks). Convergence and drop-out did not differ significantly based on the position of the opponents (χ^2^(*N* = 156, *df* = 12) = 11.62, *p* = 0.477), nor based on the combination of the position of opponents and their degree (χ^2^(*N* = 156, *df* = 6) = 7.83, *p* = 0.251).

Out of the converged networks with differing degree centrality between opponents, opponents with higher degree centrality were more likely to win (68% of the converged networks involving opponents with different degree centrality; 95% CI [51%, 81%], p_binom(40, 0.5)_ = 0.0385), whereas information centrality did not yield any significant effect on the group outcome (48% of the converged networks involving opponents with different information centrality; 95% CI [32%, 63%]; p_binom(44, 0.5)_ = 0.880). Robustness checks revealed that these degree-centrality effects were also present when considering the majority of choices in the final round of each group before convergence, drop-out, or dead-lock (see Supplement).

### Positional effects of opponents on convergence speed and polarization

**Figure 3:**
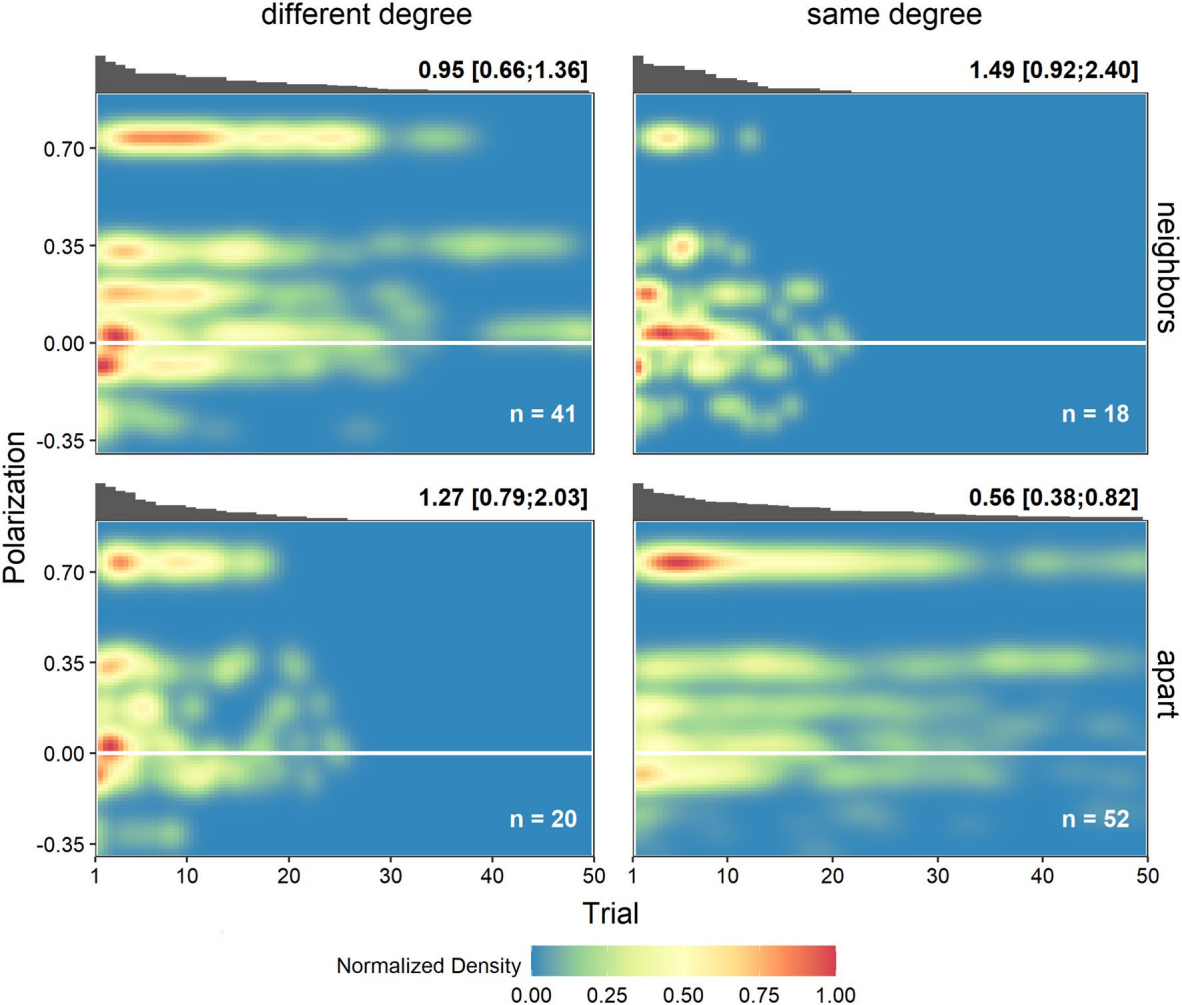
Consensus speed and degree of polarization by position in the conflict scenario. Colors of the graph represent the relative density of the polarization level of groups in the game over the course of all 50 trials with 1(red) being the highest density observed per condition. The bars above the graph indicate the proportion of groups in the respective panel not converged or dropped out in the corresponding trial (scaled between 0 and 1). Numbers above the graph state the respective hazards of convergence (i.e., convergence speed inverse), bracketed numbers are the respective 95% CI. For the definition of polarization and how patterns affect polarization, please refer to the Supplement. Densities were normalized by condition to avoid negligibly low densities across all observations and to account for uninformative differences in the number of observations per condition. Note that only a discrete number of polarization values was realized in our experiment because polarization was constrained by network configurations (the maximum polarization is 0.74, followed by 0.36; see Supplement Fig. S7).

Generally, the speed of convergence differed marginally across all seven configurations *(log-rank*-χ^2^(*N* = 156, *df* = 6) = 11.04, *p* = 0.087, for single configuration comparisons see Supplement). When collapsing conditions by centrality and neighborhood, there was a marginal effect of neighboring opponents showing that opponents being apart slows down convergence in general (*b* = – 0.431, *HR* = 0.649, *p* = 0.058; Table 1). However, this effect was qualified by the degree similarity of the individuals (Table 1): having opponents as neighbors rather than apart significantly improved speed particularly when opponents had the same degree (*b* = 0.977, *HR* = 2.657, *p* = 0.008), but not when they had different degrees (*b* = – 0.288, *HR* = 0.749, *p* = 0.413; Fig. 3). On the other hand, information centrality differences neither significantly predicted convergence speed nor significantly moderated the effects of neighbors (Table 1).

**Table 1:** Cox proportional hazards models of convergence speed in the conflict scenario.

Model	1	2a	3a	2b	3b
Neighbor (− 0.5 = neigh.) (0.5 = apart)	− 0.431+ (0.228)	− 0.327 (0.283)	− 0.344 (0.246)	− 0.449+ (0.252)	− 0.851* (0.371)
Degree sim. (− 0.5 = diff.) (0.5 = same)		− 0.175 (0.283)	− 0.183 (0.246)		
Inf. centr. sim. (− 0.5 = diff.) (0.5 = same)				− 0.043 (0.253)	− 0.074 (0.240)
Neighbor x Degree sim.			− 1.266* (0.523)		
Neighbor x Inf. centr. sim.					0.686 (0.489)
AIC	601.08	602.69	599.2	603.05	603.14
R^2^	0.023	0.025	0.059	0.023	0.035
*LR-χ*^2^(*df* = 1)	3.57 +	0.38	5.49*	0.03	1.91

SE in parenthesis. + p < 0.1; *p < 0.05.

Figure 3 additionally illustrates that networks particularly with non-neighboring, same-degree opponents were not only slow to find consensus, but also polarized to a larger extent for longer periods of time up to the maximal possible degree of polarization (one arm voting against the other arm; see Supplement for the possible patterns of polarization, also for a Figure for each of the seven opponents' positions separately).

## Discussion

Overall, groups under constrained communication are able to come to consensus in short times, while even settling competing interests within a group^13,18^. Under conflicts of interest about the group-wide decision outcome, power imbalances and relative position of opinionated opponents vis-à-vis each other can drive the speed with which conflicts are resolved and consensus is reached.

When only one member of the network was opinionated (leadership condition), networks converged quickly and mostly on the incentivized color, overall replicating the results of Gaisbauer et al.^18^. In cases of conflicting interests, more degree-central individuals were better able to wield their influence on the group, while information centrality did not seem to matter as much.

In addition, if the degrees of the opponents were the same, groups profited from direct interaction of the opponents with the unopinionated individual’s responses potentially serving as tie-breakers in voting. Generally, while individual decisions seem to require reinforcement in the local neighborhood—in line with majority rule and the “complex contagion” account of Centola^6^, overall consensus can be found more easily when dissent can be directly experienced—in line with the “small world” literature^9,29^. Thus, when coming to consensus in a group with conflicting interests, there seems to be an inherent efficiency tradeoff between the clustering of communication ties and having long ties to avoid polarization by directly interacting with opponents. Extending information propagation and leadership scenarios to situations in which varying degrees of conflicts are involved may therefore further help to reconcile two seemingly contradictive arms of research that advocate for opposing structures (clustered vs. small world designs) to achieve communication efficiency in consensus decision making.

As a cautionary note, given the very fast speed and high rate of convergence, the null-findings in the leadership scenario may be due to a ceiling effect. In addition, all non-significant results could also be due to a lack of power and thus should not be regarded as evidence for no effect. Therefore, we rather focus our interpretation on the effects found. Furthermore, the small size of our exemplary network and the small differences in centrality within may have generally limited the importance of positional effects. Accordingly, future studies should test generalizability to larger groups and to more difficult coordination tasks.

In sum, the positioning of conflicting opinions in neighborhoods that are only loosely tied to each other may be particularly susceptible to polarization—also showing the limits of a potential balancing role of uninformed group members in promoting group consensus^18,19^. Mainly visibility of invested people’s votes helps them to steer the group to their preferred outcome in cases of conflict of interests.

## Materials and methods

### Participants

Participants were recruited online via Amazon Mechanical Turk (MTurk). We ensured technically that MTurk workers did not participate multiple times or in an earlier study with a related design^18^ and data of that study are not used in the current one. Participants earned an average performance bonus of 0.35 USD (*SD* = 0.28 USD) in addition to a show-up fee (1.50 USD) and waiting bonus (up to 0.50 USD). They needed about 10 min to complete the whole study. 1596 participants in 228 networks started the main task (56.33% self-reported as female, *M*_*age*_ = 35.27 years, *SD*_*age*_ = 10.62 years). Drop-out rates after experimental assignment (7.1%) were in line with comparable online studies^18,30^. The study adhered to the Declaration of Helsinki, relevant laws, and institutional guidelines, as certified by IRB of the University of Konstanz. The University of Konstanz IRB approved the study. All participants gave informed consent.

### Procedures

The experiment was conducted online via the software oTree^31^ and the procedures are comparable to Gaisbauer et al.^18^. After informed consent, participants were presented a CAPTCHA to screen out automated scripts, followed by detailed instructions and a comprehension test.

For the main task, participants were placed in groups of 7 individuals and were instructed to find a group-wide consensus by unanimously choosing one out of two colors (blue vs. yellow) as fast as possible within 50 rounds: If the network converged on one color, everyone received a bonus fee which started at 0.50 USD and decreased by 0.01 USD increments per round. In case a participant dropped out of the study, we stopped the experiment for that group. The abandoned group members, and also all others completing the task, received a show-up fee of 1.50 USD regardless of success.

At the start of the task, each player was randomly assigned a position in the dumbbell network (Fig. 1), was given a random letter as name, and started without a pre-selected color. The network position determined which choices of other group members of the last trial were visible to whom. The task ended when all players had chosen the same color or when 50 rounds had passed without consensus. Participants received feedback on their total earnings after the main task, completed a short survey about the satisfaction with the task and potential strategies the implemented, and were debriefed. For a view of the participant screen in the task, see Fig. 1.

### Design

We varied the degree of conflict in this networked color coordination paradigm by incentivizing one or two individuals within the networks to prefer a specific color (i.e. to be opinionated; see^13,18^). In the leadership scenario, one individual (the “leader”) from each network was randomly assigned and instructed to receive an additional bonus of 0.50 USD if the network converged on a specific color. In the conflict scenario, two individuals (the “opponents”) were assigned to receive the bonus with conflicting target colors. These instructions were unknown to the other participants. The network positions of leaders and opponents were randomly determined.

### Data analysis

We used χ^2^-tests to assess how features of the network are associated with the different decision outcomes of the group (convergence, drop-out, non-convergence). To determine the role of degree and information centrality on who is more likely to win in conflict scenarios, we used binomial testing and confidence bands.

We analyzed time-until-consensus for networks with a Kaplan–Meier survival analysis with a LR-χ^2^-test using the R-package *survival*^32^. Observations were treated as right-censored if the network did not reach consensus within 50 rounds or if the network dropped out of the study. Likewise, after comparing the two scenarios, we analyzed the time-until consensus data separately for each scenario (leadership vs. conflict) to test for effects of positioning on the speed of consensus. That is, for the leadership scenario (n = 72 networks), we compared the speed of consensus between the networks with different leader positions. For the conflict scenario (n = 156), we compared the speed of consensus between all 7 configurations of how opponents can be positioned on the network. In addition, we evaluated, in a 2 × 2 design, whether consensus speed is affected by opponents being neighbors (vs. apart) and having the same (vs. different) centrality using Cox Proportional Hazard models with this information effect-coded as factors with interactions, using the R-package *survival*^32^ and LR-χ^2^-tests for testing differences between models.

We operationalized and tested positional influence of an opinionated individual by its centrality in the network with two measures: (a) degree centrality, defined as the number of direct neighbors, and (b) information centrality, a measure of the control of information flow, defined as the harmonic average of the inversely related path lengths between a focal node and all other nodes^17,20^. Please note that in this specific network set-up, more information central nodes are also the ones with higher betweenness and closeness centrality (see Fig. S1). To evaluate interactions, we used simple effects analysis of the hazards using the R package *emmeans*^33^.

The analyses and hypotheses were not preregistered. Given the number of sampled networks (72 and 156), the study is powered to detect medium effects in standard analyses with a power of 1–β = 0.80^34^.

## Supplementary Information

Supplementary Information.

## Data Availability

All data, code, and materials have been made publicly available at osf and can be accessed at https://osf.io/92pnr.
