# Supplementary material for: The role of position in consensus dynamics of polarizable networks
Source: Sci Rep. 2023 Mar 9;13:3972. doi: 10.1038/s41598-023-30613-z (PMC9998643; doi:10.1038/s41598-023-30613-z)
Supplement: Supplementary file 1 — Supplementary Information. [file 41598_2023_30613_MOESM1_ESM.pdf]

Supplement: The role of position in consensus dynamics of polarizable networks

Helge Giese, Felix Gaisbauer, Nico Gradwohl, Ariana Strandburg-Peshkin

Contents

1 Illustration of network configurations **1**

2 Robustness in consensus outcomes **3**

2.1 Choice proportions in final round 3

2.2 Survival times for all 7 conflict configurations 3

3 Polarization **3**

3.1 Polarization by possible color distributions 7

4 Sources **10**

1 Illustration of network configurations

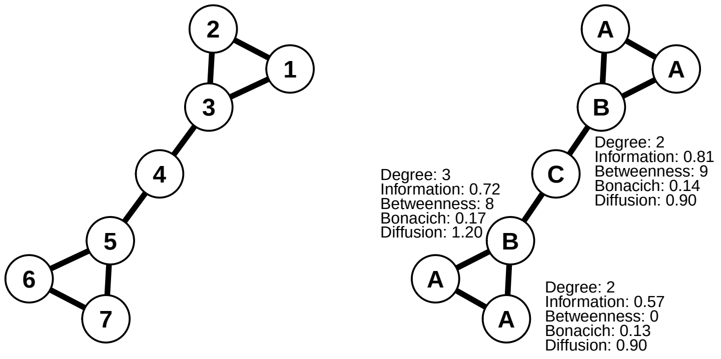

Figure S1: Description of the networks used: A network with numbered positions for reference and a network with centrality measures for each position.

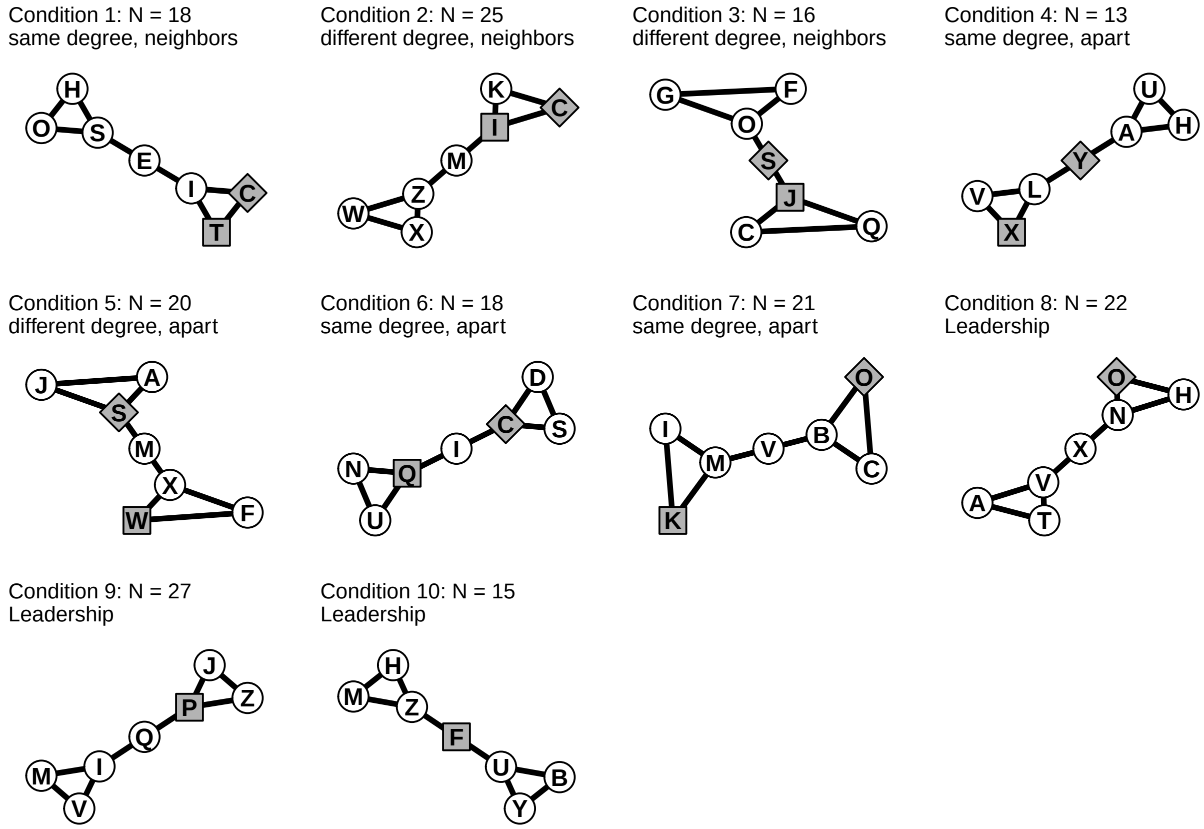

Figure S2: Example networks from the data illustrate the 10 possible distinct experimental configurations. Configurations are defined by the position of the 2 opponents (conflict, configurations 1 to 7) or the position of the leader (leadership, configurations 8 to 10).

2 Robustness in consensus outcomes

Our main conclusions are robust if we consider each of the 7 conflict configurations individually.

2.1 Choice proportions in final round

Does the benefit of higher degree hold also for non-converged networks? To address this question, we assessed the *proportion of choices in favor of the individual with the higher degree* in the final round (i.e., at convergence or before dropout) across all networks and all non-converged networks. For reference, figures also include the *proportion of blue choices* among configurations where opponents have the same degree.

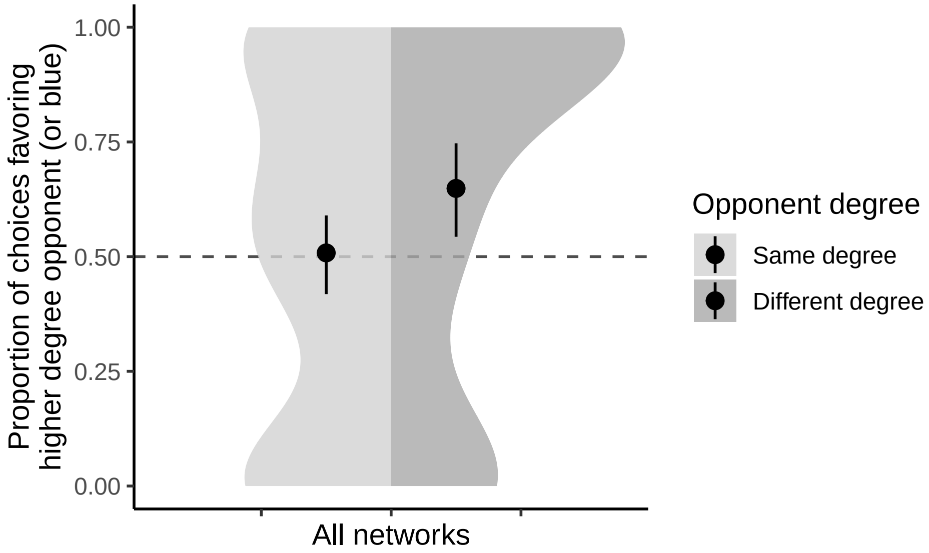

Figure S3: Distributions of the proportion color choices in favor of the higher-degree opponent across conflict configurations. For configurations without degree difference, the proportion of blue choices is shown, because no higher-degree opponent is defined.

Considering all networks, a one-sample t-test reveals that the proportion of choices in favor of the opponent with the higher degree is larger than 0.5 [Cohen's $d = 0.34$, $t(60) = 2.86$, $p = 0.006$]. The proportion of choices in favor of the opponent with the higher degree is also larger than the (arbitrary) comparison to the proportion of blue choices when both opponents have the same degree [Cohen's $d = 0.35$, $t(125.27) = 2.86$, $p = 0.047$, see Figure S3].

2.1.1 Outcome differences by information centrality

As shown in Figure S5, the opponent with higher information centrality but lower degree (condition 3) is disfavored in terms of the proportion of choices in the final round. Otherwise it is slightly favored (configurations 2, 4, and 5).

2.2 Survival times for all 7 conflict configurations

The 7 conditions marginally ($p = 0.093$) explain variance in survival times. Estimates and post-hoc tests show that condition 4 is slowest.

3 Polarization

Polarization per trial is defined as the group average of the difference between the proportion of individual agreement to directly tied neighbors and non-neighbors (individual range -1 to 1). Higher numbers indicate

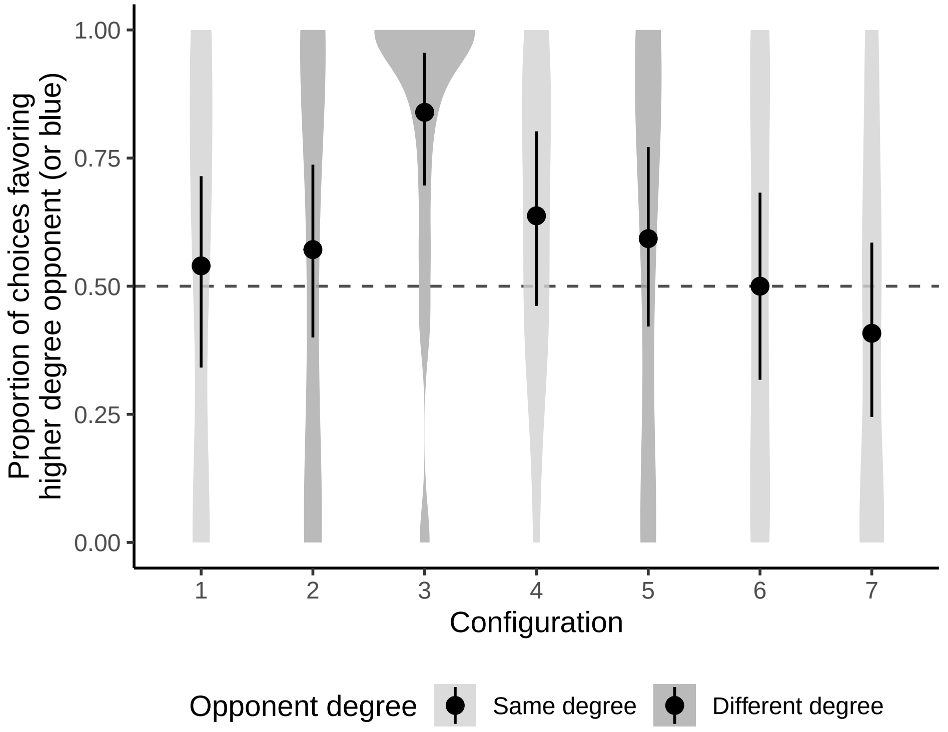

Figure S4: Distributions of the proportion color choices in favor of the higher-degree opponent for all conflict configurations. For configurations without degree difference (configurations 1, 4, 6, and 7), the proportion of blue choices is shown, because no higher-degree opponent is defined.

Table S1: Comparison of conflict configurations by means of a Cox Proportional Hazard Model.

	Dependent variable:
	Time-until-consensus
Configuration 2	−0.548 (0.386)
Configuration 3	−0.277 (0.431)
Configuration 4	−1.347* (0.593)
Configuration 5	−0.162 (0.409)
Configuration 6	−0.978* (0.440)
Configuration 7	−0.814$^+$ (0.423)
AIC	603.80
R^2	0.067
Wald Test	10.310 (df = 6)
Notes.	N = 156 networks.$^+$ $p < 0.1$; * $p < 0.05$;** $p < 0.01$; *** $p < 0.001$. Configurations were dummy-coded (reference configuration 1).

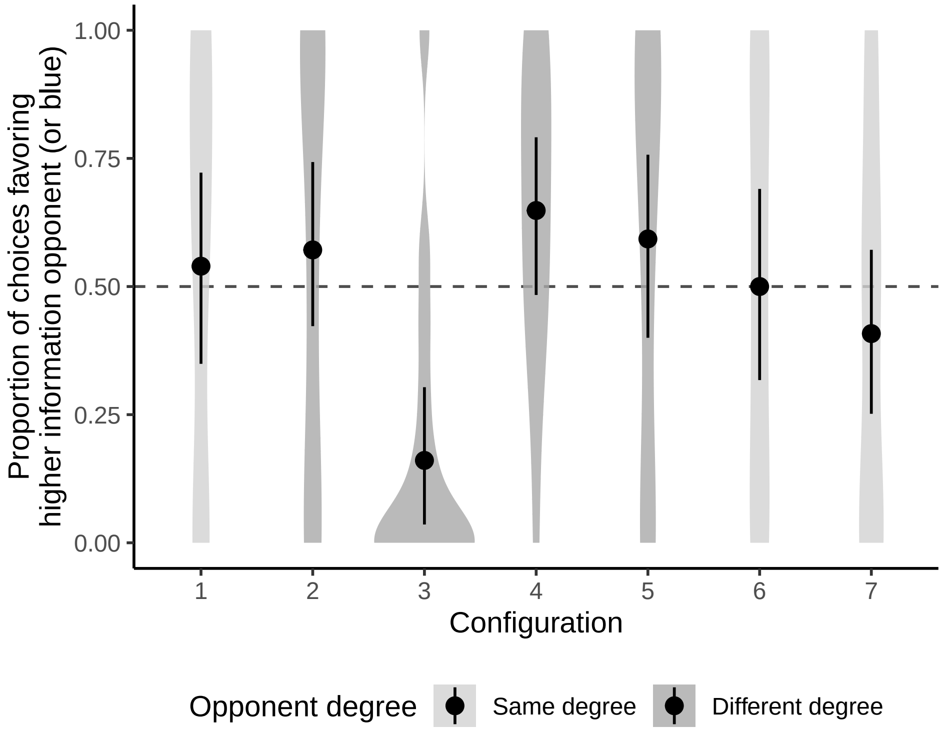

Figure S5: Distributions of the proportion color choices in favor of the higher-information centrality opponent for all conflict configurations. For configurations without differences in informaiton centrality (configurations 1, 6, and 7), the proportion of blue choices is shown, because no higher-centrality opponent is defined.

Table S2: Hazards estimated from Cox proportional hazards model for each conflict configuration.

cond_num	hazard	SE	df	asymp.LCL	asymp.UCL
Configuration 1	1.000	0.000	Inf	1.000	1.000
Configuration 2	0.578	0.223	Inf	0.271	1.232
Configuration 3	0.758	0.327	Inf	0.326	1.765
Configuration 4	0.260	0.154	Inf	0.081	0.831
Configuration 5	0.851	0.348	Inf	0.381	1.898
Configuration 6	0.376	0.165	Inf	0.159	0.891
Configuration 7	0.443	0.187	Inf	0.193	1.014

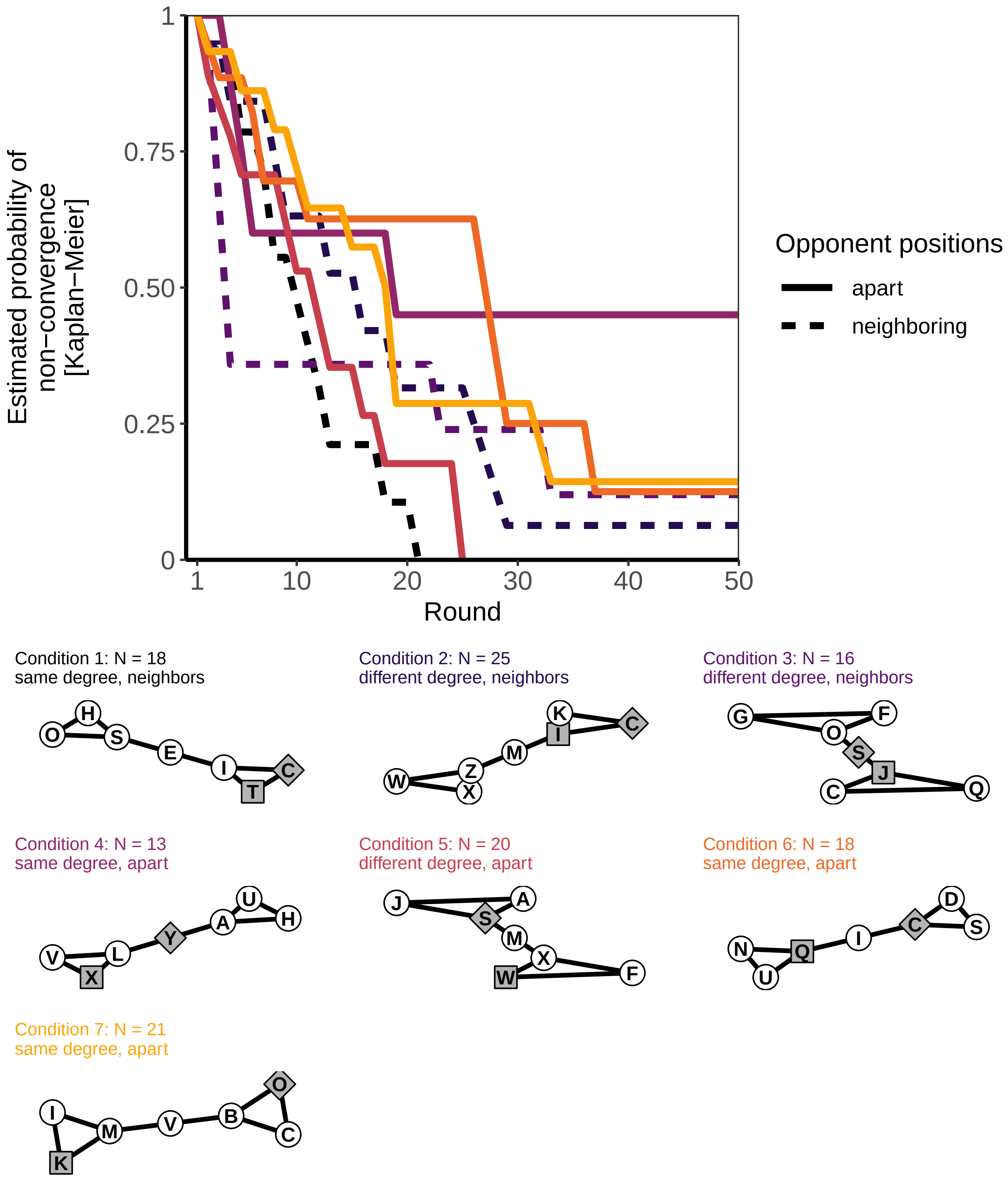

Figure S6: Survival curves for all seven conflict configurations. Correspondence of colors are outlined in the plot legend. Configurations where opponents have the same degree but are placed apart (conditions 4, 6, and 7) are slowest to converge.

Table S3: Hazard ratios by configuration: Pairwise comparisons between all conflict configurations.

contrast	ratio	SE	df	null	z.ratio	p.value
1 / 2	1.730	0.668	Inf	1	1.420	0.156
1 / 3	1.319	0.569	Inf	1	0.642	0.521
1 / 4	3.845	2.279	Inf	1	2.272	0.023
1 / 5	1.176	0.481	Inf	1	0.395	0.693
1 / 6	2.658	1.168	Inf	1	2.224	0.026
1 / 7	2.257	0.954	Inf	1	1.927	0.054
2 / 3	0.762	0.298	Inf	1	-0.695	0.487
2 / 4	2.222	1.245	Inf	1	1.425	0.154
2 / 5	0.679	0.260	Inf	1	-1.009	0.313
2 / 6	1.536	0.614	Inf	1	1.074	0.283
2 / 7	1.305	0.495	Inf	1	0.700	0.484
3 / 4	2.915	1.706	Inf	1	1.828	0.068
3 / 5	0.891	0.381	Inf	1	-0.269	0.788
3 / 6	2.015	0.883	Inf	1	1.599	0.110
3 / 7	1.711	0.716	Inf	1	1.284	0.199
4 / 5	0.306	0.180	Inf	1	-2.009	0.045
4 / 6	0.691	0.410	Inf	1	-0.622	0.534
4 / 7	0.587	0.340	Inf	1	-0.921	0.357
5 / 6	2.261	0.988	Inf	1	1.868	0.062
5 / 7	1.920	0.806	Inf	1	1.555	0.120
6 / 7	0.849	0.364	Inf	1	-0.381	0.703

less agreement among non-neighbors compared to neighbors, a higher degree of polarization.

For individual i with color choice x_{it} in trial t in group g with adjacency matrix a_{ij} and $n = 7$ individuals polarization is defined:

$$\text{Pol}_{ig}(t) = p(x_{it} = x_{jt} | j \neq i, a_{ij} = 1) - p(x_{it} = x_{jt} | j \neq i, a_{ij} = 0)$$

$$\text{Pol}_g(t) = \frac{1}{n} \sum_{i=1}^{n}$$

As illustrated in Figure S7, in our networks $\text{Pol}_{ig}(t) \in [-0.38, 0.74]$.

3.1 Polarization by possible color distributions

Possible unique color distributions. Colors yellow (y) and blue (b) can be exchanged without altering assortment or polarization. Therefore, there are 3 kinds of different situations (plus convergence without a minority):

a. 1 individual in the minority (equal 6 individuals in the majority).

b. 2 individuals in the minority (equal 5 individuals in the majority).

c. 3 individuals in the minority (equal 4 individuals in the majority).

The figure below shows all 20 distinct distributions of these individuals by increasing polarization (ignoring distributions that are equivalent for symmetry reasons).

The 20 distributions are close to perfectly correlated ($r = 0.99$) with regard to our polarization measure and assortment from the function `assortativity_nominal()` in the package `igraph`.

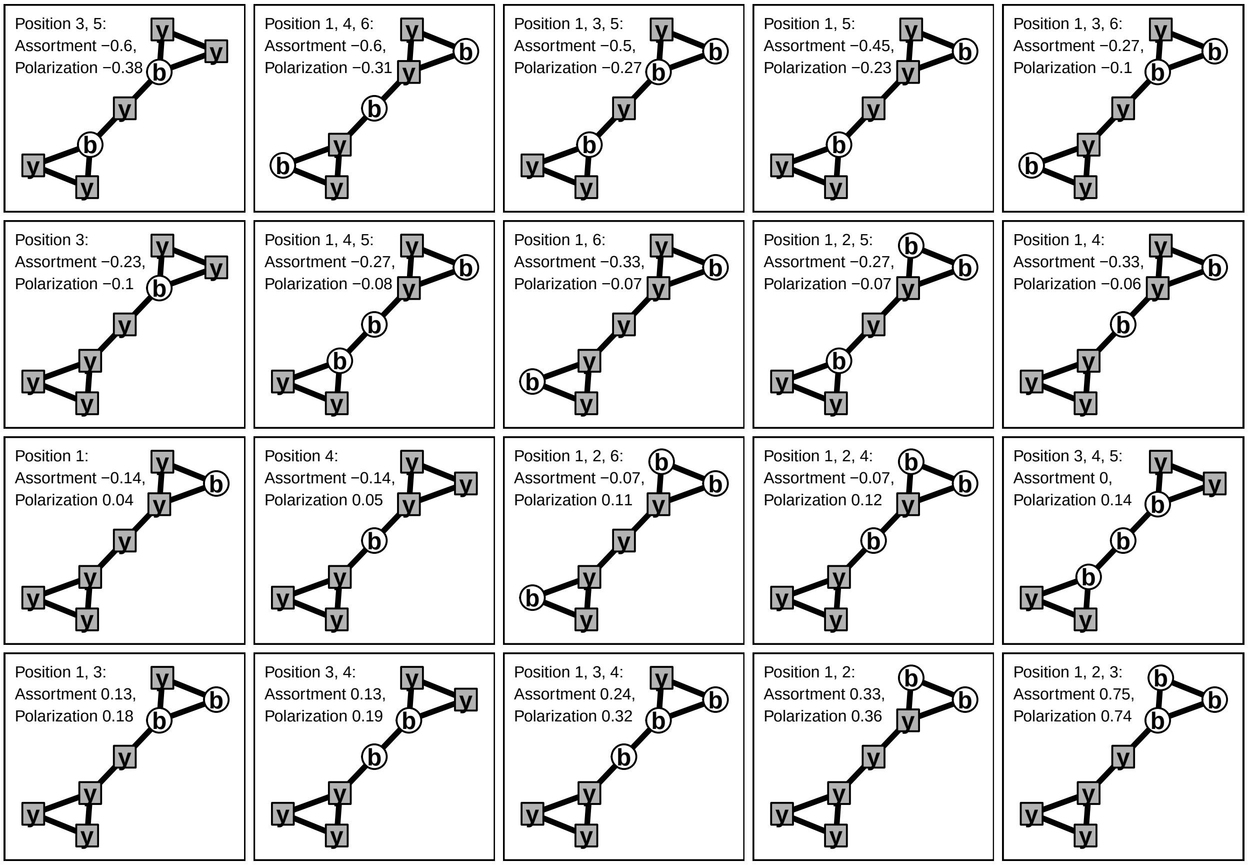

Figure S7: Possible unique color distributions with polarization and assortment.

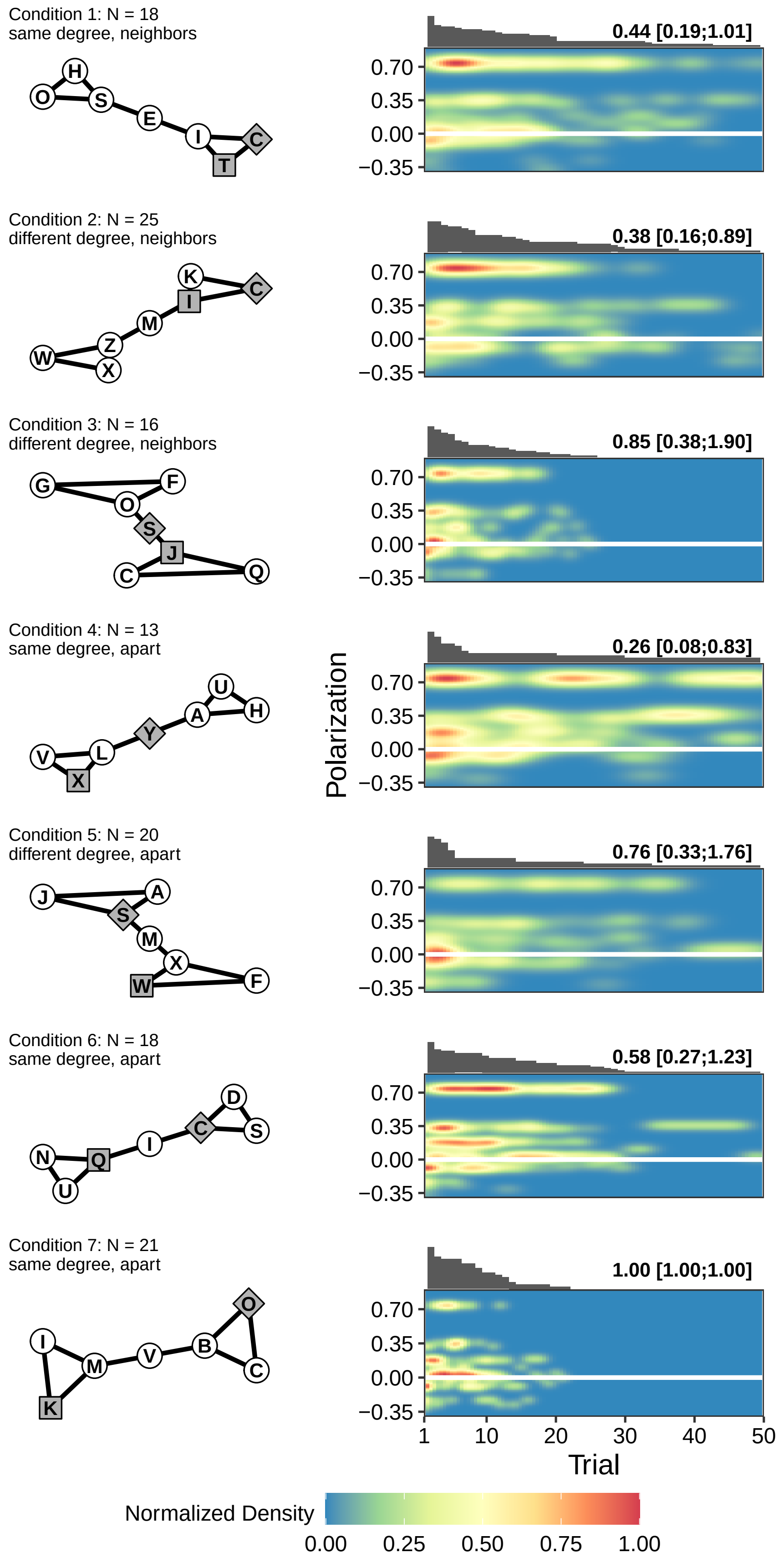

Figure S8: onsensus speed and degree of polarization by position for each configuration in the conflict scenario (see next page for notes).

Figure S8: Consensus speed and degree of polarization by position for each configuration in the conflict scenario. Colors of the graph represent the relative density of the polarization level of groups in the game over the course of all 50 trials with 1 (red) being the highest density observed per condition. The bars above the graph indicate the proportion of groups in the respective panel that was not converged or dropped out in the corresponding trial (scaled between 0 and 1). Numbers in above the graph state the respective hazards of convergence (i.e., convergence speed inverse), bracketed numbers are the respective 95%CI. Densities were normalized by condition to avoid negligibly low densities across all observations and to account for uninformative differences in the number of observations per condition. Note that only a discrete number of polarization values was realized in our experiment because polarization was constrained by network configurations (the maximum polarization is 0.74, followed by 0.36; see Supplemental Fig. S7).

4 Sources

Briatte, François. 2021. *ggnetwork: Geometries to Plot Networks with ggplot2*. https://github.com/briatte/ggnetwork.

Csardi, Gabor, and Tamas Nepusz. 2006. "The igraph software package for complex network research." *InterJournal* Complex Systems: 1695. https://igraph.org.

file., See AUTHORS. 2022. *igraph: Network Analysis and Visualization*. https://igraph.org.

Horikoshi, Masaaki, and Yuan Tang. 2018. *ggfortify: Data Visualization Tools for Statistical Analysis Results*. https://CRAN.R-project.org/package=ggfortify.

———. 2022. *ggfortify: Data Visualization Tools for Statistical Analysis Results*. https://github.com/sinhrks/ggfortify.

Kassambara, Alboukadel, Marcin Kosinski, and Przemyslaw Biecek. 2021. *survminer: Drawing Survival Curves using ggplot2*. https://rpkgs.datanovia.com/survminer/index.html.

Lenth, Russell V. 2022. *emmeans: Estimated Marginal Means, aka Least-Squares Means*. https://github.com/rvlenth/emmeans.

R Core Team. 2021. *R: A Language and Environment for Statistical Computing*. Vienna, Austria: R Foundation for Statistical Computing. https://www.R-project.org/.

Tang, Yuan, Masaaki Horikoshi, and Wenxuan Li. 2016. "ggfortify: Unified Interface to Visualize Statistical Result of Popular R Packages." *The R Journal* 8 (2): 474–85. https://doi.org/10.32614/RJ-2016-060.

Terry M. Therneau, and Patricia M. Grambsch. 2000. *Modeling Survival Data: Extending the Cox Model*. New York: Springer.

Therneau, Terry M. 2021. *survival: Survival Analysis*. https://github.com/therneau/survival.

Wickham, Hadley. 2016. *ggplot2: Elegant Graphics for Data Analysis*. Springer-Verlag New York. https://ggplot2.tidyverse.org.

Wickham, Hadley, Winston Chang, Lionel Henry, Thomas Lin Pedersen, Kohske Takahashi, Claus Wilke, Kara Woo, Hiroaki Yutani, and Dewey Dunnington. 2021. *ggplot2: Create Elegant Data Visualisations Using the Grammar of Graphics*. https://CRAN.R-project.org/package=ggplot2.

Wickham, Hadley, Romain François, Lionel Henry, and Kirill Müller. 2021. *dplyr: A Grammar of Data Manipulation*. https://CRAN.R-project.org/package=dplyr.

Wilke, Claus O. 2020. *cowplot: Streamlined Plot Theme and Plot Annotations for ggplot2*. https://wilkelab.org/cowplot/.

Xie, Yihui. 2014. "knitr: A Comprehensive Tool for Reproducible Research in R." In *Implementing Reproducible Computational Research*, edited by Victoria Stodden, Friedrich Leisch, and Roger D. Peng. Chapman; Hall/CRC. http://www.crcpress.com/product/isbn/ 9781466561595.

———. 2015. *Dynamic Documents with R and knitr*. 2nd ed. Boca Raton, Florida: Chapman; Hall/CRC. https://yihui.org/knitr/.

———. 2021. *knitr: A General-Purpose Package for Dynamic Report Generation in R*. https://yihui.org/knitr/.
